# Locking *versus* Non-locking Neutralization Plates with Limited Excision and Internal Fixation for Treatment of Extra-articular Type a Distal Tibial Fractures

**DOI:** 10.2174/1874325001711010057

**Published:** 2017-02-28

**Authors:** Kai-hua Zhou, Nong Chen

**Affiliations:** Department of Orthopedic Surgery, Qingpu Branch of Zhongshan Hospital of Fudan University, Qingpu District, Shanghai, China

**Keywords:** AO/OTA type A, Distal tibial fracture, Locking plate, Neutralization plate, Non-locking plate

## Abstract

**Purpose:**

This study aimed to compare the clinical, radiologic, and cost-effectiveness results between locking and non-locking plates for the treatment of extra-articular type A distal tibial fractures.

**Methods:**

We performed a retrospective review of AO/OTA 42-A1, A2 distal tibial fractures treated by plates from January 2011 to June 2013. Patients were divided to the locking plate group or the non-locking plate group. Clinical outcomes, radiographic outcomes, and hospitalization fee were compared between the two plates groups.

**Results:**

28 patients were treated with a locking plate and 23 patients were treated with a non-locking plate. The mean follow-up was 18.8 months (12-23 months). There were no significant differences between the groups in surgical time, bleeding, bone union time, or AOFAS scores. The cost of the locking plate was ¥24,648.41 ± 6,812.95 and the cost of the non-locking plate was ¥11,642 ± 3,162.57, p < 0.001. Each group had one patient that experienced superficial infection these wounds were readily healed by oral antibiotics and dressing changes. To date, five patients in the locking group and ten patients in the non-locking group had sensations of metal stimulation or other discomfort (X^2^ = 3.99, p < 0.05) Until the last follow-up, 14 patients in the locking plate group and 18 patients in the non-locking plate group had their plates removed or wanted to remove their plates (X^2^ = 4.31, p < 0.05).

**Conclusion:**

The use of locking or non-locking plates provides a similar outcome in the treatment of distal fractures. However the locking plate is much more expensive than the non-locking plate.

## BACKGROUND

Fractures of the distal third of the tibia are common. They represent about 3-10% of all tibial fractures [1]. Intramedullary nail fixation for most fractures is still the gold standard [2], but if the marrow cavity is too small or the fracture line is near the joint surface, we just only can use the plates to to treat the fractures. AO/ASIF type 42-A1 and A2 fracture of the distal tibia is a simple fracture. According to the AO principles of fracture management, simple fracture needs anatomical reduction, strong fixation, absolute stability, and primary healing. The traditional technique of open anatomic reduction and internal fixation of distal tibial fractures requires extensive soft-tissue dissection and often leads to subsequent periosteal injury. High rates of complications, including postoperative infection, delayed union, and non-union, have been reported [3, 4]. Using a limited incision, minimally invasive technology can be a very good solution to these problems [2, 5], reducing both iatrogenic soft tissue injury and damage to the blood supply. In recent years, locking plates techniques have gained popularity among orthopedic surgeons which can preserve the periosteal blood supply and increase stability [7-11]. Kim found that in the setting of elderly ankle fractures, locking plates are at least equivalent biomechanically to standard plates, but Minihane got different conclusions that the posterolateral antiglide plate demonstrated improved biomechanical stability as compared to the lateral locking plate in osteoporotic bone [12, 13]. And Edward also found that the one-third tubular construct was equivalent to locking plate constructs with respect to union, post-operative range of motion, and rates of complications, but the locking plate was much more expensive [14]. Up to now, sufficient evidence to favor one plate over the other in the treatment of distal tibial simple fractures is also lacking in the current literature. In the present study, we evaluated whether there were clinical, radiologic, and hospitalization fee between the use of locking and non-locking neutralization plates for treating AO type 42-A1, A2 fractures of the distal tibia with the limited incision minimally invasive technology.

## MATERIALS AND METHODS

We performed a retrospective review of 70 patients with distal tibia AO type 42-A1, A2 fractures from January 2011 to June 2013. The mechanisms of injury were sprains, high falling injuries, and traffic accidents. Patients were included in this study if they were age 18 or older that underwent surgical management with non-locking plates or locking plates as neutralization plates with limited excision and MIPPO technique. The exclusion criteria of this study were open fractures, polytrauma, pathologic fractures, patients younger than 18 years. The study was approved by the ethics review committee of the our hospital and written informed consent was obtained from all patients. Finally, a total of 51 patients were included in this study. Patients were subsequently divided into two groups according to the type of osteosynthesis used. The locking plates group (n = 28), a titanium locking compression distal tibial plate (LCP, Trauson, China) was used. The non-locking plates group (n = 23) comprsed patients in which a dynamic compression distal tibial plate (DCP, Trauson, China) was used. The two groups were similar with respect to age, gender distribution, fracture patterns according to the AO classification system and the preoperative waiting time. Twenty-nine patients underwent surgical treatment within 6-8 hours after injury; others with soft tissue swelling or merging diseases underwent selective operation (Table **1**). The surgeries were performed by two senior attending doctors.

### OPERATION PROCEDURE

Under general or spinal anesthesia, the patient was positioned supine on a radiolucent table. With a thigh tourniquet inflated after exsanguination, routine preparation and draping of the injured limb was performed. A 3-5 cm incision was made over the fracture, then the hematoma and soft tissue in the fracture site were cleaned without periosteal stripping. The fracture was reduced under direct vision, then bone forceps or Kirschner wire were used to reduce and hold the fracture temporarily. One or two cortical screws were used as lag screws to compress the fracture site. Proper length anatomical locking or non-locking plates were selected according to the fracture line. According to the plate location, two 2-4 cm longitudinal incisions were made in the skin beneath the two ends of the plate. One incision was at the midline of the medial malleolus, the other was made along the medial aspect of the tibia located at the proximal end of the plate. An extraperiosteal, subcutaneous tunnel could then be fashioned between these two incisions using blunt dissection. The great saphenous veins were protected and the plate was inserted percutaneously from the distal to proximal direction. Four screws were inserted distally and three or four screws were inserted proximally. If the patient also had a fibular fracture to be fixed, the posterolateral approach was selected to fix the fibular fracture first.

Physical rehabilitation with active motion of the ankle joints was initiated on the second postoperative day. Partial weight bearing was recommended 8-12 weeks after surgery; full weight bearing was recommended 3-4 months after surgery according to the union of the fracture. Clinical evidence of infection was recorded. Deep infections were defined as those below the deep investing muscular fascia. Superficial infections were clinically confined to the dermal and subcutaneous tissues.

Follow-up assessments were included clinical and radiographic examinations once a month until the fracture had unified. Solid union was defined as the visualization of cross trabeculations on the AP and lateral radiographs. Clinical outcomes were assessed using the American Orthopaedic Foot and Ankle Society (AOFAS) ankle-hind foot instrument one year after surgery. The hospitalization fee were identified including labour costs, radiographs, surgery fee, anesthesia fee, implants cost, pharmacy supplies and hospital resoures. We also noted patients who had the plate removed because of local pain and/or skin irritation related to plates and screws.

Data analyses were performed using SPSS 18.0. To determine the significance of intergroup differences, the T test and Fisher’s exact test were used. Statistical significance was defined as p < 0.05 and all statistical analyses were reviewed by an independent statistician.

## RESULTS

**Fig. (1):**
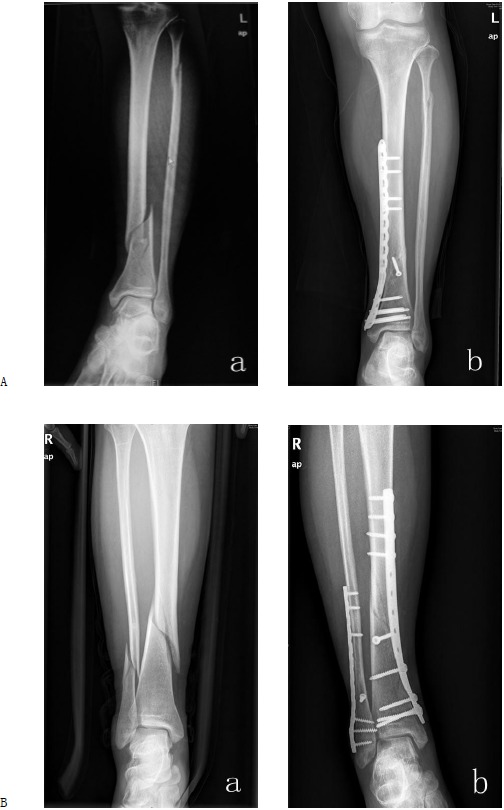
The X-ray images of typical plating cases. **(A)** a 47-year-old female with Type A1 left distal tibia fracture and proximal fibula fracture due to a traffic accident. a: The prooperative radiographs is shown; b: The fracture was treated with a locking plate (LCP). The fracture healed 4 moths after the operation. **(B)** a 44-year-old female with Type A1 right distal tibial fracture and fibula fracture due to a traffic accident. a: the preoperative radiographs is show; b: The fractures were treated with two non-locking plates (DCP) for the tibial and fibula. The fractures healed 3 months after the operation.

The mean follow-up time was 18.8 months (range, 12-23months). In the locking plate group, the surgical time was 62.44 ± 18.81 min, surgical bleeding was 77.60 ± 24.62 ml, bone union time was 13.71 ± 2.01 weeks, and the mean AOFAS score was 88± 2.01. In the non-locking plate group, the surgical time was 68.97 ± 21.31 min, surgical bleeding was 87.07 ± 30.63 ml, bone union time was 14.26 ± 2.02 weeks, and the mean AOFAS score was 86± 1.73. All fractures were primary healing. There was no significant difference between the groups in surgical time, bleeding, bone union time, or AOFAS scores. The total hospitalization fee except implants cost but including labour costs, radiographs, surgery fee, anesthesia fee, pharmacy supplies and hospital resouresin the locking plate groups was ¥12,135 ± 1,035.65 per case, and ¥12,030 ± 987.55 in the non-locking plate groups, P > 0.05.The locking plate implants cost was ¥24,648.41 ± 6,812.95 while the non-locking plate implant cost was ¥11,642 ± 3,162.57, p < 0.001. One patient in each group experienced superficial infection; these wounds were healed with the use of oral antibiotics and regular dressing changes. To date, Implant removal was necessary in 14 cases (50%) and 18 (78%) cases in the locking plate group and non-locking plate group because of local pain and/or skin irritation related to plates and screws. The need of implant removal was significantly higher in the non-locking plate group than in the locking plate group (X^2^ = 4.31, p < 0.05) . The cost of the removal of the plate is similar in each patients no matter he treated by locking plate or non-locking plate (P > 0.05) (Table **2** and Fig. **1**).

## DISCUSSION

The AO/ASIF type 42-A1 and A2 fracture of the distal tibia is a simple fracture. According to the AO principles of fracture management, simple fractures need anatomical reduction, strong fixation, absolute stability, and primary healing. A bridge-plating technique in simple fractures to achieve relative stability may prolong the union time and as well as the time to full weightbearing; it may also cause other negative outcomes [6]. The fractures in our study were reduced and fixed with lag screws. Neutralization plates were placed across the fracture to augment fracture fixation. Plate fixation helps to limit bend, rotation, and axial loads across the fracture site. Limited excision and internal fixation with MIPPO technique produces significantly less disruption of soft tissue and extraosseous blood supply than traditional surgical methods [15] and can also protect the blood supply, promote fracture healing, and reduce complications. The technique may be performed with either a locking plate or non-locking plate. A locking plate provides stability as a fixed-angle construct; fixed-angle properties obviate the need for compression and contact between plate and bone. A non-locking plate obtains fixation stability by the frictional force between plate and bone. This compression can cause disturbances in blood supply to the bone and can introduce an unfavourable condition for bone union. In theory, the locking plate has obvious clinical advantages to non-locking plate. However, Takemoto reported that both styles of plates resulted in equivalent results when they were used as a neutralization device in a cadaveric study [16]. Sachiyuki also reported that there were no differences in bone union rate or complications when locking and non-locking plates were used for neutralization in malleolar fractures [17]. Similarly, our study got equivalent results in fracture healing time and complications. This result contrasts with the study by Ufuk *et al*. [18]. These authors reported that the time to achieve bone union was longer in their locking plate group (17.2 *vs*. 13.1 weeks). The difference between the two studies may be caused by different fixation techniques. We used limited excision and internal fixation with neutralization plates fixed with a lag screw, but Ufuk *et al*. used the percutaneous minimally invasive bridge-plated technology. This difference demonstrates once again that a bridge-plating technique in simple fracture patterns to achieve relative stability may prolong the union time. We emphasize here that simple fractures need absolute stable fixation and primary healing. In our study, surgical time and bleeding is less in the locking plate group. This may be caused by that the locking screw is a self-tapping screw. This can simplify the surgical process and reduce the bleeding when we tapped the bone. But there was no statistical difference.

The question of whether the fibula also needs to be fixed is always controversial. Hazaika *et al*. [7] reported that the fibula should be fixed in cases where it was deemed necessary to restore the stability and normal anatomy of the ankle joint, or where it was considered helpful to have a “template” for length. Toms *et al*. [19] and Strauss *et al*. [20] also advised to fix the fibula. Hasenboehler *et al*. [6] and Oh *et al*. [21] did not need fibular fixation to achieve satisfactory fracture reduction and alignment; postoperative failure of stability was not observed in their patients. In our clinical experience, if the distal fibula fracture involved the ankle and the mortise is abnormal, the fibula needs to be fixed to prevent traumatic arthritis and postoperative joint instability. If the fibula fracture is located in the middle, the fracture needs open reduction and fixation only to ensure tibial anatomical reduction; the upper fibula fractures does not need to be fixed.

The postoperative infection rate of distal tibial fractures is 0-4.4% [6, 7, 22, 23]. Because the distal tibia is covered only by a thin layer of soft tissue, it tends to have a higher incidence of infection, especially when the stabilizing plate is placed on the medial side of the distal tibia [7, 22, 24]. The infection rate with the percutaneous minimally invasive bridge-plate technique is much lower [2]. In our study, there were no cases of deep infection, but each group had a single patient with superficial infection; these infections were both successfully treated with oral antibiotics and dressing changes. These results are similar to other research [2, 6, 7, 22]. Because our study shows that the infection rate is similar with the outcomes of the percutaneous minimally invasive bridge-plate technique, this suggests that the additional incision above the fracture does not increase the risk of postoperative infection.

The need of implant removal was significantly higher in the non-locking plate group than in the locking plate group. The hospitalization fee to remove the plates were similar in the locking plate group and non-locking plate group. So the cost is not the influence factor to the patients whether to remove the plates. The only reason to the patients to remove the plates is the local pain and/or skin irritation related to plates and screws The reason we think maybe that the locking plate construction is actually thinner than the non-locking plate (2.4 mm *vs*. 4.0 mm), And the soft tissue and skin is thinner on the medial side, it is more likely to feel plate stimulation or other discomfort, especially in the lean bodies. The locking plate also has its shortcomings. It was not exactly fit to the bony contour, especially in the region of the medial malleolus; the patients often felt discomfort and the gap between plate and bone may facilitate hematoma and obscure the hidden danger of infection.

There was no significant difference between the two groups in the AOFAS scores. Gao *et al*. [25] treated distal tibial metaphyseal fractures with polyaxial locking plates in patients that had a mean AOFAS score of 87.3. Our patients had similar scores. That means that limited excision and internal fixation with MIPPO technique for the treatment of extra-articular distal tibial fractures with either locking or non-locking plates can get excellent clinical results.

Our results examined the cost of both types of plates. The cost was much higher in the locking plate group (p < 0.05). The total hospitalization fee except the implants cost in the locking plate groups was ¥12,135 ± 1035.65 per case, and ¥12,030 ± 987.55.There was no significant difference. But the locking plate implant cost was ¥24,648.41 ± 6,812.95 while the non-locking plate implant cost was ¥11,642 ± 3,162.57, which were significantly different, p < 0.001, Even though the clinicle and radiographic outcome was almost the same, with the increase in cost of plates implant, locking plates greatly increase the economic burden placed upon patients

There were several limitations in our study. First, the cases were retrospective review and not randomly selected. Second, the number of patients (sample size) was relatively small. Third, the implant choice may be influenced a particular surgeon’s own biases as it is not clear exactly why the decision was made to use a specific plant in each case.

## CONCLUSION

Limited excision and internal fixation with locking or non-locking plates provides a similar outcome in the treatment of distal tibial fractures of AO type A1, A2. Anatomical reduction and lag screws are recommended for stable fixation and early healing. The locking plate is much more expensive, but the locking plate seems only superior with respect to the need for implat removal that could justify its higher cost.

## Figures and Tables

**Table 1:** Patient demographic data.

	Locking plate group	Non-locking plate group	X^2^/t	P value
Men/women	20/8	15/8	X^2^ = 0.226	0.63
Mean age(y)	50.4(22~77)	49.3(31~68)	t = 0.92	0.36
AO classification			X^2^ = 0.017	0.9
A1	19	16		
A2	9	7		
The mechanisms of injury			X^2^ = 2.81	0.42
sprain	5	3		
traffic accident	18	15		
crush	5	3		
high falling injuries	0	2		
Fibular fracture			X^2^ = 0.18	0.98
Proximal 1/3	12	11		
Middle 1/3	3	2		
Distal 1/3	7	5		
Emergency surgery	15	14	X^2^ = 0.27	0.60
Preoperative waiting time(day)	1.85(0~4)	1.96(0~5)	t = 0.73	0.44

**Table 2:** Clinical outcomes(x±s).

Group	N	Surgical time (min)	Bleeding (ml)	Bone union (week)	AOFAS score	Cost (¥)
Locking plate	28	62.44 ± 18.81	77.60 ± 24.62	13.71 ± 2.01	88 ± 2.01	24648.41 ± 6812.95
Non-locking plate	23	68.97 ± 21.31	87.07 ± 30.63	14.26 ± 2.02	86 ± 1.73	11642.00 ± 3162.57
T	-	1.77	1.63	0.88	0.51	14.61
P	-	0.06	0.10	0.34	0.56	<0.001
